# Unconsciously Triggered Emotional Conflict by Emotional Facial Expressions

**DOI:** 10.1371/journal.pone.0055907

**Published:** 2013-02-07

**Authors:** Jun Jiang, Kira Bailey, Antao Chen, Qian Cui, Qinglin Zhang

**Affiliations:** 1 Faculty of Psychology, Southwest University, Beibei, Chongqing, China; 2 Key Laboratory of Cognition and Personality (Southwest University), Ministry of Education, Beibei, Chongqing, China; 3 Department of Psychological Sciences, University of Missouri, Columbia, Missouri, United States of America; Macquarie University, Australia

## Abstract

The present study investigated whether emotional conflict and emotional conflict adaptation could be triggered by unconscious emotional information as assessed in a backward-masked affective priming task. Participants were instructed to identify the valence of a face (e.g., happy or sad) preceded by a masked happy or sad face. The results of two experiments revealed the emotional conflict effect but no emotional conflict adaptation effect. This demonstrates that emotional conflict can be triggered by unconsciously presented emotional information, but participants may not adjust their subsequent performance trial-by trial to reduce this conflict.

## Introduction

Every day we are confronted by a plethora of information, some of which may be conflicting. To optimize our behavior, we minimize the effects of this conflict. Cognitive conflict and the mechanisms of control have been examined extensively, and researchers have broadened this work to the affective domain [1] by using various paradigms, such as the emotional Flanker task [2], the emotional Stroop task [3], [4], the Word-Face Stroop task [5], [6], [7] and the emotion AX-Continuous Performance Task [1], [8]. These studies generally show that the reaction times (RTs) on emotional incongruent trials are significantly longer than those on emotional congruent trials [1], [2], [8]. In addition, research has also revealed that RTs on emotional incongruent trials are longer when preceded by an emotional congruent trial than when preceded by an emotional incongruent trial; likewise RTs on emotional congruent trials are shorter when preceded by an emotional congruent trial than when preceded by an emotional incongruent trial [e.g.], [ 7,9]. This effect is similar to the conflict adaptation effect in the cognitive control literature. These findings suggest that participants tend to adjust their subsequent performance to reduce conflict after they have experienced emotional conflict.

Prior work has provided evidence for emotional conflict and emotional conflict adaptation effect, but has not directly assessed whether these effects can be triggered by conscious and unconscious emotional information. The experimental paradigms used in the previous studies were designed to examine emotional conflict induced by conscious emotional information. For example, in the Word-Face Stroop task [5], [6], [7], [9], [10], words are overlaid upon faces and participants are instructed to judge the emotional valence of the words [5], [6] or vice versa [7], [9]. Participants are further instructed to ignore the irrelevant stimulus [7], but both the word and face are presented long enough for the participant to perceive and process consciously. Therefore, the emotional conflict in previous studies may stem from interference between conscious processing of the task-relevant and task-irrelevant emotional stimuli simultaneously.

In the domain of cognitive control, the data have consistently suggested that cognitive conflict can be triggered by unconscious cognitive information, although there is a lively debate on unconscious cognitive conflict adaptation [11], [12] [13], [14], [15]. Using a masked prime target paradigm, researchers demonstrated an unconscious conflict effect, similar to the conscious cognitive conflict effect. In contrast, the data for an unconscious conflict adaptation effect have been mixed, with many studies failing to demonstrate the effect [e.g. 11], [14], while others have observed trial-by-trial conflict adaptation effects [12], [15], [16]. Moreover, the unconscious conflict adaptation effect was not a result of low-level response or stimulus repetition priming effects [15].

Related to the current investigation, works using the affective priming paradigm have demonstrated that subliminal emotional information can impact subsequent performance [17], [18], [19], [20]. For instance, Murphy and Zajonc [17] found that when participants were exposed to a briefly presented (4 ms) happy or angry face followed by affectively neutral Chinese ideograph, they evaluated the ideograph as more positive when the ideograph was primed by a happy face than when it was primed by an angry face. The affective priming paradigm originally developed by Fazio, Sanbonmatsu, Powell, and Kardes [21], has been compared to other classic conflict tasks such as the Stroop and Flanker tasks [19], because the RTs for categorization usually vary as a function of congruency between prime and target valence [22]. Therefore, this paradigm seems particularly well-suited for the current investigation examining unconscious emotional conflict and conflict adaptation effects.

The purpose of the current study was to determine whether emotional conflict and emotional conflict adaptation could be triggered unconsciously. To achieve this goal, the backward-masked affective priming task was used, which is similar to the classic affective priming paradigm with the addition of a backward mask immediately following the prime. In order to enhance the unconscious emotional conflict, facial expressions were used as primes and targets instead of words [3]. Moreover, due to the fact that attention is a critical factor in processing the masked primes [15], [23], a brief fixation was used to increase the amount of attentional resources available for processing the masked prime. Two experiments were conducted in this study. Due to differences between the masked affective priming task used in the current study and the classic emotional conflict task used in previous work, a control condition with supraliminal primes was included in each experiment in order to establish that emotional conflict and control occurs in the masked affective priming paradigm. Experiment 1 was designed to demonstrate the basic unconscious and conscious emotional conflict effect, while Experiment 2 was designed to investigate the emotional conflict adaptation effect. Based on the findings in the literature on unconscious cognitive control and subliminal affective priming, two predictions were made: 1) unconscious emotional conflict would be observed because of generic emotional valence incompatibility effects; that is, the RTs and error rates on emotional incongruent trials should be higher than those on emotional congruent trials; 2) unconscious emotional conflict adaptation should be found if participants are inclined to reduce the emotional conflict they experience. Taken together, these experiments provide further evidence of the automatic nature and flexibility (reflected by unconscious emotional conflict adaption) of emotional information processing. Furthermore, exploring unconscious emotional conflict and control are important steps toward understanding the function of unconscious emotion.

## Experiment 1

### Materials and Methods

#### Ethics Statement

This procedures and analyses was approved by the ethics committee of Southwest University of China. Informed consent was obtained from all participants after the explanation of the experimental protocol according to the principles expressed in the Declaration of Helsinki.

### Participants

Forty-four undergraduate students (36 females) between 20 and 23 (*M* = 21.34, *SD* = 1.27) years of age at Southwest University in China participated for monetary remuneration. All participants were right-handed with normal or corrected-to-normal vision, and had no history of head injury or physical and mental illness. This study was approved by the local ethics committee of Southwest China University, and written informed consent was obtained from all participants after the explanation of the experimental protocol.

#### Apparatus and materials

Stimuli were presented against a black background at the center of a 17-inch View-Sonic CRT monitor (frequency 70 Hz, resolution 1024×768) with the E-prime 1.1 software package (Psychology Software Tools, Pittsburgh, PA).

Thirty-six sad facial expressions and thirty-six happy facial expressions were selected from the new version of the Chinese Facial Affective Picture System (CFAPS) [24]. Twelve sad and 12 happy facial expressions served as primes, and the remaining as targets. Each group of expressions was balanced between males and females. Sad (*M* = 3.12, *SD* = 0.60) and happy (*M* = 6.67, *SD* = 0.42) facial expressions differed significantly (*t*(35) = 34.00, *p*<0.001) in valence. Mean arousal ratings for sad (*M* = 5.35, *SD* = 1.21) and happy (*M* = 5.30, *SD* = 0.90) facial expressions were matched (*t*(35) = −0.251, *p* = 0.803). Emotional ratings for primes and targets are presented in Table 1. In addition, practice materials were taken from the old version of CFAPS. The mask was a checkerboard pattern of one of the facial expressions created by using the Photoshop filter called scramble (www.telegraphics.com.au/sw/info/scramble.html). Participants viewed the stimulus from a distance of 70 cm. The entire prime or target subtended a visual angle of 8.82°×9.26°(260 by 300 pixels).

**Table 1:** Emotional ratings (*M*±*SD*) for primes and targets.

	Prime		Target	
	Positive (n = 12)	Negative (n = 12)	Positive (n = 24)	Negative (n = 24)
Valence (1–9)	6.72±0.46	2.98±0.42	6.65±0.41	3.19±0.67
Arousal (1–9)	5.31±0.97	5.75±1.27	5.29±0.89	5.15±1.15

#### Design

Participants were randomly and equally assigned to either the strongly masked group (formal experimental group) or the weakly masked group (control experimental group). Prime-target pairs were divided into 2 categories based on whether or not the prime valence and target valence matched: congruent and incongruent. Prime and target faces were either positive or negative in valence. Thus, 4 conditions were created: 1) positive congruent trials; 2) positive incongruent trials; 3) negative congruent trials; 4) negative incongruent trials. Each prime face was presented twice in non-consecutive trials, while each target face was only presented once in each valence congruency condition. This resulted in a total of 96 prime-target pairs in each test block. All trials were randomly presented.

#### Procedure

**Figure 1:**
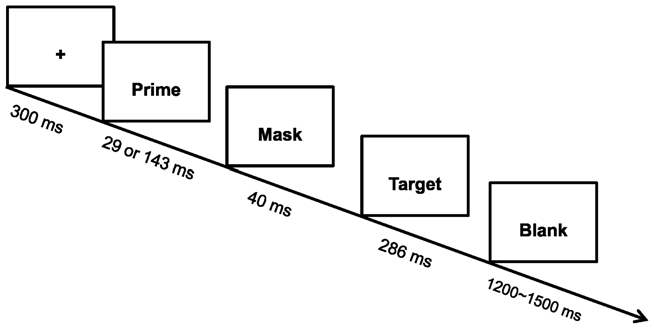
Schematic representation of the experimental procedure.

Written informed consent was obtained from all participants. At the beginning of each trial, a fixation cross was presented for a duration of 300 ms, followed by the appearance of a prime face for 29 ms (2 frames, in the strongly masked group) or 143 ms (10 frames, in the weakly masked group), and then by a mask for 40 ms. After the backward mask offset, a target face was presented for 286 ms (20 frames) followed by a blank for a random duration ranging between 1200 and 1500 ms (see Figure 1). Participants were instructed to respond as quickly and accurately as possible by pressing the key on the keyboard to indicate the target valence. Half of the participants were instructed to press “F” with the left index finger if the target valence was positive and to press “J” with the right index finger if the target valence was negative; the finger-to-key mapping was reversed in the remainder of the participants.

Participants completed 16 full-feedback practice trials, followed by 4 test blocks of 96 trials each. Finally, participants completed a two-alternative forced-choice discrimination task on the primes. Participants were asked to take a 2-minute break with eyes closed after they performed each block. In the discrimination task, the sequence and timing of the stimuli were exactly the same as in the test blocks, except that the target was replaced by a response screen instructing the participants to indicate as accurately as possible whether a happy or a sad face was presented and that the finger-to-key mapping was the same as the test blocks [15], [25]. Before administrating the discrimination task, participants were also told that the discrimination task had no time pressure and that frequency of happy and sad faces was equal. When all tasks were over, participants were asked if they could clearly see the masked prime faces.

#### Data analysis

Incorrect trials and trials that fell outside three standard deviations of the mean RTs were excluded from analysis for each participant in each condition [22]. All trials following errors were also excluded from the RT analyses. Mean RTs and error rates of weakly masked and strongly masked groups were separately submitted to two repeated measures ANOVAs, with target valence (positive vs. negative) and emotional congruency (congruent vs. incongruent) as within subject variables. A significance level of 0.05 was used for all statistical tests.

### Results and Discussion

#### Prime discrimination

None of the participants reported that they had clearly recognized the facial expressions before the mask in the strongly masked group. In contrast, accuracy rates of prime identification in the weakly masked group were high due to the supraliminal nature of the primes. Two statistics were used to examine the visibility of the masked prime valence [25]. In the strongly masked group, the accuracy rate of the forced-choice task in the ranged from 42% to 54% and averaged 49.27% (*SD* = 0.06) and did not differ significantly from chance, *t*(21) = −0.541, *p* = 0.595. The participants had relatively low hit rates (*M* = 49.04%, *SD* = 0.12) and false alarm rates (*M* = 50.76%, *SD* = 0.13), and the one sample t-test revealed that the *d′* score (*M* = −0.39, *SD* = 0.34) was not significantly larger than zero, *t*(21) = −0.534, *p* = 0.599. In the weakly masked group, the discrimination accuracy ranged from 56% to 88% (*M* = 69.32%, *SD* = 0.09), and was greater than chance, *t*(21) = 10.63, *p*<0.001. The *d′* score (*M* = 1.07, *SD* = 0.55) was significantly larger than zero, *t*(21) = 9.17, *p*<0.001, with relatively high hit rates (*M* = 70.32%, *SD* = 0.12) and false alarm rates (*M* = 31.91%, *SD* = 0.13). These results demonstrate that the participants were unable to recognize the valence of masked primes consciously in the strongly masked condition.

#### Emotional conflict analysis

**Figure 2:**
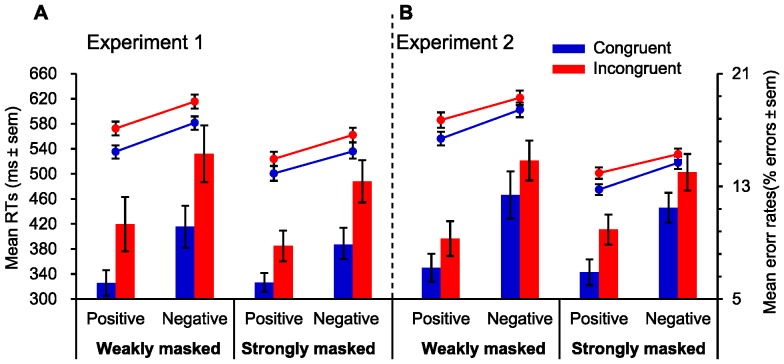
Mean RTs for correct trials and mean error rates as a function of target valence and valence congruency in weakly masked and in strongly masked groups. (A) Emotional conflict results in Experiment 1. (B) Emotional conflict results in Experiment 2. Emotional conflict effects were significant on mean RTs and error rates for positive and negative conditions in the weakly masked and strongly masked groups. Points in the line chart represent the mean RTs in each experimental condition. Bar graphs represent the mean error rates in each experimental condition. Error bars depict standard errors of the means (SEM).

Figure 2A illustrates the mean RTs and error rates as a function of target valence and valence congruency in the weakly masked (left panel) and strongly masked (right panel) groups. In the weakly masked group, the repeated measures ANOVA on RTs revealed a robust main effect of target valence, *F*(1,21) = 70.50; *p*<0.001, and of valence congruency, *F*(1,21) = 77.45; *p*<0.001, indicating that participants responded to negative faces (*M* = 599 ms, *SD* = 49.66) significantly slower than to positive faces (*M* = 554 ms, *SD* = 47.88), and that they responded considerably slower on incongruent trials (*M* = 594 ms, *SD* = 50.84) than on congruent trials (*M* = 559 ms, *SD* = 45.21), respectively. There was no significant (*F*<1) interaction between target valence and emotional congruency. For the analysis of the error rates, the main effects of target valence, *F*(1,21) = 10.62; *p* = 0.004, and of valence congruency, *F*(1,21) = 8.92; *p* = 0.007, were significant, but the interaction between them not significant (*F*<1). Further analysis suggested that the mean error rate was higher when the target valence was negative (*M* = 12.91%, *SD* = 0.07) than when it was positive (*M* = 8.50%, *SD* = 0.06), and that the mean error rate in the incongruent condition (*M* = 13.05%, *SD* = 0.08) was higher than in the congruent condition (*M* = 8.32%, *SD* = 0.05). The results of mean RTs and error rates analysis together demonstrate an emotional conflict effect.

In the strongly masked group, the repeated measures ANOVA on RTs revealed a robust main effect of target valence, *F*(1,21) = 46.80; *p*<0.001, and of valence congruency, *F*(1,21) = 36.60; *p*<0.001, indicating that participants responded to negative faces (*M* = 549 ms, *SD* = 55.47) significantly slower than to positive faces (*M* = 512 ms, *SD* = 59.91), and that they responded considerably slower on incongruent trials (*M* = 543 ms, *SD* = 52.54) than on congruent trials (*M* = 518 ms, *SD* = 61.40), respectively. There was no significant (*F*<1) interaction between target valence and emotional congruency. For the analysis of the error rates, the main effects of target valence, *F*(1,21) = 11.98; *p* = 0.002, and of valence congruency, *F*(1,21) = 11.91; *p* = 0.002, were significant, suggesting that the mean error rate was higher when the target valence was negative (*M* = 11.11%, *SD* = 0.05) than when it was positive (*M* = 7.48%, *SD* = 0.03), and that the mean error rate in the incongruent condition (*M* = 11.07%, *SD* = 0.04) was higher than in the congruent condition (*M* = 7.52%, *SD* = 0.05). The results of the strongly masked group echoed the results of weakly masked group, demonstrating the existence of the emotional conflict effect even when the primes were presented below conscious awareness.

In line with our predictions, the typical emotional conflict effect was found. Specifically, these results clearly indicated that no matter whether target valence was positive or negative, there was a robust unconscious emotional conflict effect evidenced by longer RTs and higher error rates on incongruent than on congruent trials. Experiment 2 was conducted to explore whether unconscious emotional conflict would modulate performance on subsequent trials just as conscious emotional conflict does. Experiment 2 also served as a test of replication for Experiment 1 and allowed us to examine conflict and adaptation (i.e., control) in one experiment.

## Experiment 2

### Materials and Methods

#### Ethics Statement

Same as Experiment 1.

#### Participants

Sixty-two undergraduate students (26 females) between 19 and 24 (*M* = 22.30, *SD* = 1.23) years of age at Southwest University of China participated for monetary remuneration. All participants were right-handed with normal or corrected-to-normal vision, and had no history of head injury or physical and mental illness.

#### Apparatus and materials

These were identical to Experiment 1.

#### Design and procedure

Participants were randomly and equally assigned to either the strongly masked (formal experimental group) group or the weakly masked group (control experimental group). Based on the crossing of previous trial type (congruent vs. incongruent) and current trial type (congruent vs. incongruent), four conditions were created: 1) congruent–congruent (CC); 2) congruent–incongruent (CI); 3) incongruent–congruent (IC); 4) incongruent–incongruent (II) [9]. All trials were pseudo-randomly presented to ensure that the proportions of the above four categories were equally presented across the trials. There were 4 test blocks of 97 trials each. Within each test block, each condition consisted of 24 trials. Furthermore, each target face only appeared twice in each block, once in each emotional congruency condition, and none of the target faces appeared in two consecutive trials in order to avoid stimulus repetition priming effects. Therefore, it was not necessary for us to take stimulus repetitions into consideration. Based on whether the response keys for the previous trial and the current trial were the same or not, trials could be divided two categories: response change trials and response repetition trials. This manipulation allows us to test whether trial-by-trial sequence modulation effects were truly due to regulatory changes in emotional conflict control or whether they merely reflected lower-level response repetition priming effects [15].

#### Data analysis

Except the mentioned in the following, all data handling process same as Experiment 1. Trials following errors and the first trial in each test block were also excluded. To test the reliability of Experiment 1, mean RT and error rate data were again submitted to repeated measures ANOVAs similar to Experiment 1. To explore the emotional conflict adaption effect, mean RTs and error rates in the weakly masked and strongly masked groups were separately submitted to two repeated measures ANOVAs with previous trial type (congruent vs. incongruent), current trial type (congruent vs. incongruent) and response type (response change, response repetition) as within subject variables.

### Results and Discussion

#### Prime discrimination

Brief questioning after the experiment revealed that none of the participants in the strongly masked group could clearly recognize the emotional valence of the masked prime face. We again used two statistics to test the visibility of the masked prime valence [25]. The mean accuracy rate of the forced-choice prime discrimination task was 50.48% (*SD* = 0.07) and was not significantly different from chance, *t*(30) = 0.40, *p* = 0.692. Participants again had relatively lower hit rates (*M* = 60.21%, *SD* = 0.17) and false alarm rates (*M* = 59.41%, *SD* = 0.16), and the one sample t-test suggested that the *d′* score (*M* = 0.01, *SD* = 0.37) was not significantly larger than zero, *t*(30) = 0.17, *p* = 0.866.

In contrast, in the weakly masked group, the discrimination accuracy ranged from 56% to 81% (*M* = 70.30%, *SD* = 0.08), and was greater than chance, *t*(30) = 14.86, *p*<0.001. The *d′* score (*M* = 1.16, *SD* = 0.50) was significantly larger than zero, *t*(30) = 12.74, *p*<0.001, with relatively high hit rates (*M* = 71.58%, *SD* = 0.13) and false alarm rates (*M* = 30.94%, *SD* = 0.14). The results are consistent with Experiment 1 and again demonstrate that the participants were unable to recognize the valence of masked primes consciously in the strongly masked condition.

#### Emotional conflict analysis

In the weakly masked group, the analysis on RTs yielded that the RTs differed significantly depending on emotional congruency, *F*(1, 30) = 44.94; *p*<0.001, indicating that the RTs in the emotional incongruent condition were longer (*M* = 604 ms, *SD* = 64.38) than in the emotional congruent condition (*M* = 579 ms, *SD* = 59.81). The main effect of target valence was also significant, *F*(1, 30) = 46.68; *p*<0.001, indicating that the RTs were faster for positive faces (*M* = 571 ms, *SD* = 64.33) than for negative face (*M* = 612 ms, *SD* = 62.98). A significant interaction between emotional congruency and target valence was found, *F*(1, 30) = 4.85; *p* = 0.035). Further analysis suggested that the emotional conflict effect, *t*(30) = 2.18; *p* = 0.037, was significantly larger when target valence was positive (*M* = 30 ms, *SD* = 24.85) than when it was negative (*M* = 19 ms, *SD* = 23.07). Error rates were significantly higher, *F*(1, 30) = 8.14; *p* = 0.008, in the emotional incongruent condition (*M* = 12.26%, *SD* = 0.06) than in the emotional congruent condition (*M* = 10.06%, *SD* = 0.06), and participants committed more errors (*F*(1, 30) = 11.80; *p*<0.001) when they recognized negative faces (*M* = 13.71%, *SD* = 0.08) than when they recognized positive faces (*M* = 8.18%, *SD* = 0.05). However, the interaction was not significant (*F*<1) (see Figure 2B, left panel).

In the strongly masked group, the RTs differed significantly depending on emotional congruency, *F*(1, 30) = 89.35; *p*<0.001, indicating that the RTs in the emotional incongruent condition were longer (*M* = 521 ms, *SD* = 53.75) than in the emotional congruent condition (*M* = 500 ms, *SD* = 53.12). The main effect of target valence was also significant, *F*(1, 30) = 66.58; *p*<0.001, revealing that the RTs were faster for positive faces (*M* = 488 ms, *SD* = 51.80) than for negative face (*M* = 525 ms, *SD* = 51.53). A significant interaction between emotional congruency and target valence was found, *F*(1, 30) = 7.80; *p* = 0.009). Further analysis indicated that the emotional conflict effect, *t*(30) = 2.77; *p* = 0.010, was significantly larger when target valence was positive (*M* = 26 ms, *SD* = 16.86) than when it was negative (*M* = 14 ms, *SD* = 17.56)(see Figure 2b). The error rates data were analyzed in the same fashion as the RTs data. Error rates were significantly higher, *F*(1, 30) = 12.05; *p* = 0.002, in the emotional incongruent condition (*M* = 11.97%, *SD* = 0.05) than in the emotional congruent condition (*M* = 9.19%, *SD* = 0.04), and participants committed more errors (*F*(1, 30) = 10.73; *p* = 0.003) when they recognized negative faces (*M* = 12.74%, *SD* = 0.06) than when they recognized positive faces (*M* = 8.42%, *SD* = 0.05). However, the interaction between them was not significant (*F*<1) (see Figure 2B, right panel).

#### Emotional conflict adaptation analysis

**Figure 3:**
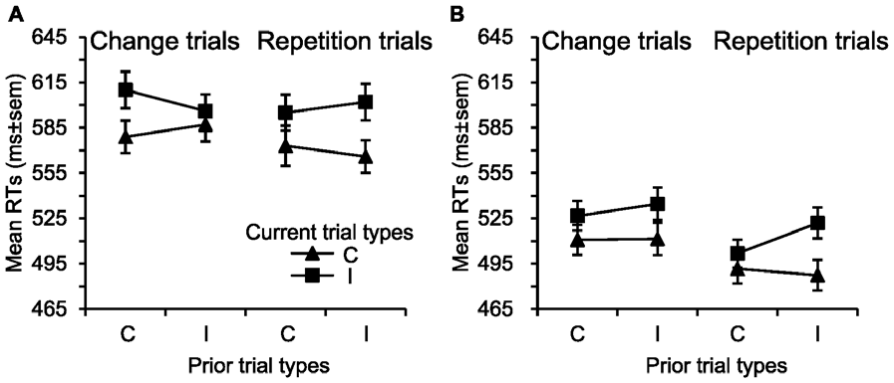
Mean RTs for correct trials as a function of previous trial types for response change trials and response repetition trials in the weakly masked and strongly masked groups. (A) Emotional conflict adaptation results in the weakly masked group. (B) Emotional conflict adaptation results in the strongly masked group. The classic emotional conflict adaptation effect was observed in the weakly masked group. The endpoint of the line chart depicts the mean RTs in each experimental condition. Error bars represent the standard error of the mean (SEM). C = Congruent, I = Incongruent.

The analysis of the RTs in the weakly masked group revealed that the three-way interaction among prior trial type, current trial type and response trial type was significant, *F*(1, 30) = 26.62; *p*<0.001. This suggested the existence of trial-by-trial modulation effect. Two separate two-way ANOVAs on the RTs further suggested that the interaction between previous trial type and current trial type was significant in response change trials, *F*(1, 30) = 20.63; *p*<0.001, and response repetition trials, *F*(1, 30) = 6.44; *p* = 0.017, but, as Figure 3a illustrated, the direction of modulation effects were reversed in response change and response repetition trials. In response change trials, the classic conflict adaptation effect was observed. That is, RTs in II trials (*M* = 596 ms, *SD* = 62.47) were faster than in CI trials (*M* = 610 ms, *SD* = 67.78), *t*(30) = −4.55, *p*<0.001, while the RTs in IC trials (*M* = 587 ms, *SD* = 61.78) were slower than in CC trials (*M* = 579 ms, *SD* = 59.56), *t*(30) = 2.31, *p* = 0.028. The ANOVAs for error rates in the weakly masked condition did not yield a significant three-way interaction (*F*<1) (see Figure 3A).

In the strongly masked condition, the ANOVA on RTs suggested that a trial-by-trial modulation effect was obtained, as evidenced by a significant three-way interaction among previous trial type, current trial type and response type, *F*(1, 30) = 4.96; *p* = 0.034. Two separate two-way ANOVAs on the RTs suggested that the interaction between previous trial type and current trial type was significant in response repetition trials, *F*(1, 30) = 20.89; *p*<0.001, but not in response change trials, *F*(1, 30) = 1.72; *p* = 0.200. Further analysis on the interaction in response repetition trials revealed that the RTs in II trials (*M* = 522 ms, *SD* = 56.62) were slower than in CI trials (*M* = 502 ms, *SD* = 51.83), *t*(30) = 5.75, *p*<0.001, while no significant difference was found on the RTs between IC trials (*M* = 487 ms, *SD* = 56.69) and CC trials (*M* = 492 ms, *SD* = 53.97), *t*(30) = 1.12, *p*>0.05. Although a sequence modulation effect was found, the direction was just reverse to the classic conflict adaptation effect (see Figure 3b). Therefore, the sequence modulation effect observed in this experiment was caused by response priming, not by emotional conflict. The ANOVAs for error rates indicated that the three-way interaction among prior trial types, current trials types and response types was not significant, *F*(1, 30) = 2.61; *p*>0.05. Overall, no significant emotional conflict adaption effect was found in the strongly masked condition (see Figure 3B).

Although the trials were pseudo-randomly presented in this experiment, the emotional conflict effect was again observed in strongly masked and weakly masked conditions, which replicates the results of Experiment 1.However, in Experiment 2 the emotional conflict effect was larger when the targets were positive faces. This discrepancy may arise from the way in which the trials were presented. In contrast with the random presentation in Experiment 1, participants may have formed expectancies about the upcoming masked face when the trials were pseudo-randomly presented as in Experiment 2. Furthermore, due to the fact that negative faces can attract more attention than positive faces even when they are unconsciously presented (for a review, see [23]), it is possible that the negative prime faces received deeper processing than the positive prime faces. Consequently, under emotional incongruent conditions, the emotional conflict effect should be larger when the targets are negative faces than when they are positive faces.

Experiment 2 failed to provide evidence for the emotional conflict adaptation effect in the strongly masked condition. Although in this condition a sequence modulation effect was found [19], further analysis indicated that it was a result of low level response priming, not emotional conflict. However, an emotional conflict adaptation effect was observed in the conscious condition, suggesting that unlike emotional conflict, conscious awareness is required for conflict adaptation to occur.

## General Discussion

To our knowledge, the current study is among the first to examine unconscious emotional conflict and control. Using a backward-masked affective priming paradigm, we demonstrated that unconscious emotional information could initiate emotional conflict as evidenced by longer RTs and higher error rates in the emotional incongruent condition relative to the emotional congruent condition. However, the data did not support the presence of the emotional conflict adaptation effect. That is, the emotional conflict effect reflected on both RTs and error rates in trial n was not significantly reduced relative to trial n-1. These findings indicated that even though the conflict-inducing stimuli were presented unconsciously, the primes interfered with processing the target when the valence of the prime and target was incongruent; however, the experience of unconscious emotional conflict did not lead to a more cautious response strategy and adjustment of subsequent behavior to reduce the unconscious emotional conflict.

### Unconscious emotional conflict

One explanation for the experience of conscious emotional conflict states that the emotion of both the relevant and irrelevant stimuli is automatically perceived; when the emotion present in the stimuli does not match, processing of the irrelevant stimulus interferes with processing the relevant stimulus [1]. This explanation may also be plausible for unconscious emotional conflict. In the present two experiments, participants were not consciously aware of the valence of the masked prime, but may still have processed the emotional information of the prime unconsciously, allowing the prime to interfere with processing of a later consciously presented target. There is neuropsychological evidence to support this assumption (for a review, see [23]). Using backward-masking paradigms as in the current experiments, previous studies have demonstrated that masked faces evoked enhanced neural responses, such as the P3 or late positive potential, even though the participants did not report conscious awareness of the faces [26], [27], [28]. Furthermore, the association between facial expressions and emotional valence is relatively automatic (e.g., smiling with positive affect, frowning with negative affect) [1], [8], and this may increase the likelihood that a negative or positive prime presented unconsciously would lead an individual to expect that target valence should be the same as the prime valence. On incongruent trials where the target valence is not consistent with the individual's expectation, the prime valence would interfere with judgment of the target valence, resulting in increased RTs and error rates. The latter notion fits well with the response competition theory [29], which posits that after the prime is rapidly presented participants build an expectation that the target will have the same valence as the prime. If they are incongruent, participants have to revise their expectations and thus delay the response.

### Unconscious emotional conflict adaptation

The null effect of emotional conflict adaptation is not consistent with previous studies, which demonstrated that individuals will strategically adjust their emotional information processing to reduce conflict [7]. This disparity may lie in the way that task-irrelevant emotional conflict-inducing stimuli are presented. In previous studies, emotional conflict-eliciting stimuli and emotional target stimuli were presented simultaneously for a long duration of time, while in the current study the emotional conflict-eliciting stimuli were unconsciously presented before the emotional target stimuli for a brief time followed by a mask. The design of the current studies was meant to make conscious identification of the irrelevant stimuli difficult. The results are in line with most studies on unconscious cognitive conflict adaptation, in which a significant unconscious conflict adaptation effect was not observed [e.g. 11], [14]. These studies concluded that if participants did not perceive the conflict in trial n-1 consciously, then they could not regulate information processing to reduce conflict in trial n. In short, if conflict is never consciously detected by the individual, no conflict adaptation effect would occur [30]. These studies also underlined the importance of the strength of the neutral trace of trial n-1 in bringing about unconscious conflict adaptation. If the neural traces were very weak and/or had died out before the trial n was presented, then it would be unlikely that the individual would modify their performance on trials n based on conflict experience in trial n-1. In fact, evidence from other studies have confirmed that compared with conscious information processing, brain activation invoked by unconscious information processing is weaker [e.g. 31] and decays faster [e.g. 32]. This explanation seems plausible in the current experiments in which the intervals between trials may be long enough to let the neutral traces of conflict disappear, resulting in the absence of conflict adaptation effects.

In conclusion, the present study demonstrated that emotional conflict could be triggered by unconsciously presented faces, but this unconscious emotional conflict was not strong enough to elicit emotional conflict adaptation. Further studies on unconscious emotional conflict and unconscious emotional conflict adaptation will be essential for deepening our understanding of emotional conflict mechanisms and the implications of the automatic nature of emotional processing. One avenue for future work would be to examine the effect of shortening the delay between the prime and target to reduce the possibility of conflict information decay. Future studies might also examine the effect of primes just slightly above chance identification. This could provide insight regarding the point at which emotional conflict adaptation becomes possible in this task. Furthermore, investigations using ERPs or fMRI techniques will be necessary for revealing the neural correlates of unconscious emotional conflict and control.
